# Cloning and functional analysis of the promoter of a stress-inducible gene (*Zmap*) in maize

**DOI:** 10.1371/journal.pone.0211941

**Published:** 2019-02-08

**Authors:** Bo Jin, Zunlai Sheng, Ishfaq Muhammad, Jianqing Chen, Hongliang Yang

**Affiliations:** 1 College of Veterinary Medicine, Northeast Agricultural University, Harbin, P. R. China; 2 Heilongjiang Key Laboratory for Animal Disease Control and Pharmaceutical Development, Harbin, P. R. China; Birla Institute of Technology and Science, INDIA

## Abstract

The anionic peroxidases play an important role in a variety of plant physiological processes. We characterized and isolated the *Zmap* promoter (P*Zmap*) at the 5′ flanking region in order to better understand the regulatory mechanisms of *Zmap* gene expression. A series of P*Zmap* deletion derivatives, termed a1 –a6, at positions −1694, −1394, −1138, −784, −527 and −221 from the translation start site were blended to the β-glucuronidase reporter gene. *Agrobacterium*-mediated transformation method was used to study each deletion construct in tobaccos. Sequence analysis showed that several *cis*-acting elements (MYB binding site, Box-II, a TGACG-element, a CGTCA-element and a low temperature responsive element) were located within the promoter. Deletion analysis suggested the sequence between −1,694 and −1394bp may contain *cis*-elements associated with GUS up regulation. The MYB binding site (-757) might act as a negative drought-responsive element. There might be repressor elements located in the region (−1,694 to −1394bp) to repress *Zmap* expression under 4°C. The characterized promoter would be an ideal candidate for genetic engineering for improving the resistance of maize to different stressors.

## Introduction

Plants are usually subjected to many hostile environments, such as drought, salinity, and low temperatures, which severely affect plant growth and productivity. A series of complex cell signal transduction processes will occur to limit the damage under these abiotic conditions. Many kinds of defense mechanisms can also be activated under abiotic stressors; among them, the expression of resistance genes can be found under single or multiple stress conditions. Therefore, the expression of stress-inducible genes and promoters play an important role in plant resistance.

Promoters are important DNA sequence signals in gene expression. Constitutive promoters can continuously drive transcription and expression of downstream genes, including exogenous genes. However, gene over expression may hinder the energy required for normal growth and the synthesis of RNA and proteins [1–3]. In contrast, inducible promoters limit gene expression to specific tissues or organs, or to defined growth stages, such as limited growth conditions or the presence of insults, which could reduces the adverse effects on plant growth. Therefore, the study of inducible promoters will improve our understanding the molecular mechanisms of signaling pathways [4].

Anionic peroxidases play an important role in a variety of plant physiological processes such as lignifications, suberifications, wound repair, and defense against disease [5]. Anionic peroxidase expression can be divided into constitutive and inducible expression. Inducible expression anionic peroxidases considered some of the most important plant protective isozymes, play an important role in both pathogen infection and abiotic stressors [6]. To understand the expression mechanism of the anionic peroxidase gene, we functionally characterized the promoter region of anionic peroxidase in maize (*Zmap* promoter). In this report, we investigated the *Zmap* promoter region inducible activity and identified the response of the 5’-flanking sequence to different stimuli, including methyl jasmonate (MeJA), low temperature and drought. The study could provide valuable insights into the mechanism of the *Zmap* promoter involved in *Zmap* gene expression patterns under abiotic stressors.

## Materials and methods

### Plant materials and growth conditions

*Zea mays* (B73) plant seeds were collected in the experimental field of our university and the author was not obliged to have any permissions. This work did not involve endangered or protected species and the species *Zea mays* (B73) is a common plant. Maize plants were propagated within a controlled environment chamber with a photoperiod of 16 h light/8 h dark at 25°C. At the same time, tissue-culture tobacco *Nicotianatabacum* (NC 89) plants were raised on Murashige-Skoog (MS) medium supplemented with 30 g/l sucrose, 7 g/l agar, 3 mg/l 6-benzyladenine, 0.2 mg/l α-naphthaleneaetic acid and adjusted to pH 5.8. Plants were maintained 16 h light/8 h dark photoperiod at 25°C. Genetic transformation experiments were carried out with fully developed tobacco leaves.

### Promoter cloning and sequence analysis

Bioinformatics analysis to identify putative regulatory motifs in the *Zmap* promoter sequences from maize was performed using the database of PlantCARE [7].

For determination of the structure of *Zmap* promoter, polymerase chain reaction (PCR) was carried out using the primer pair aP-F/aP-R shown in Table 1 with maize DNA as template. Cycling conditions including 94°C for 5min; 30 cycles of 94°C for 45s, 58°C for 40s, 72°C for 2min; and 72°C for 10min. BamHI and NcoI restriction enzymes restriction sites were represented by underlined letters. Recombinant clones were sequenced following cloning of PCR products into the pMD18-T vector.

**Table 1:** Primers used for polymerase chain reaction (PCR).

Primer name	Primer sequence (5'- 3')
aP-F(a-1)	CGGGATCCTGCCGTGATACCGACTTGA
a2	CGGGATCCAACTCACAGCACCTACGCAC
a3	CGGGATCCATACCCACACCACCCACCAC
a4	CGGGATCCCCTGATTCCCCATCTGTGTG
a5	CGGGATCCAATAGCCCAGTTGCCATCTC
a6	CGGGATCCGAGAAATGAGATCATCCCACC
aP-R	CCCATGGTTCAGCTTGCTTGTTGCTTG
ACTIN- F	CGGAATTCACAATATCGGTTCCGCTGC
ACTIN- R	CCCATGGCTTCTTATTCGATCAGAC
GUS-F	CGGGATCCTGCCGTGATACCGACTTGA
GUS-R	CCCATGGTTCAGCTTGCTTGTTGCTTG

### Genetic transformation and construction of expression vectors

**Fig 1:**
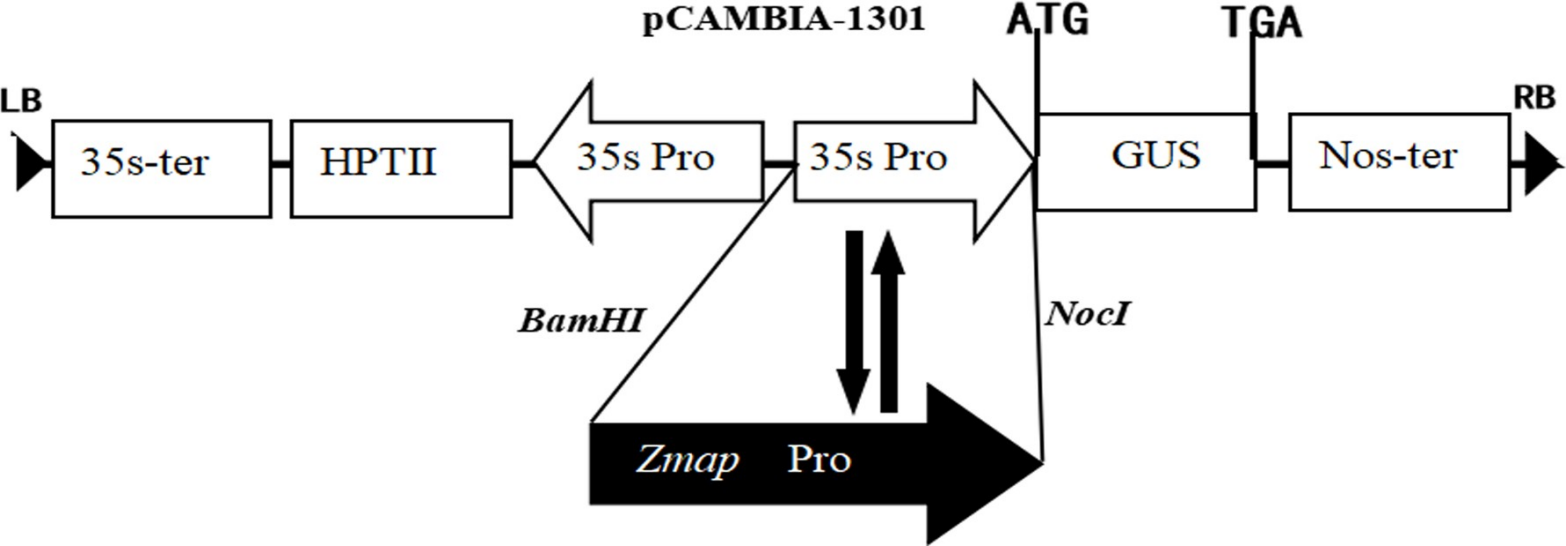
Schematic representation of the P*Zmap*: GUS construct. The insertion position of the *Zmap* promoter in the vector is indicated with restriction enzyme sites (BamHI and NcoI). LB, left border; RB, right border; *35s-ter*; Cauliflower Mosaic virus 35S terminator; *35s* Pro, Cauliflower Mosaic virus 35S promoter; *GUS*; β-glucuronidase gene; *HPTII*, hygromycin phosphotransferase (II) coding region; *NOS-ter*, nopaline synthase terminator; *Zmap* Pro, *Zmap* promoter.

The functional regions of the *Zmap* promoter were investigated by5’-end deletion analysis. A series of *Zmap* promoter deletions were generated by PCR, named as a1 (1694 bp), a2 (1394 bp), a3 (1138 bp), a4 (784 bp), a5 (527 bp) and a6 (221 bp) using the primers shown in Table 1. CaMV35S promoter was replaced following the cloning of amplicons into the pCMBIA1301 plasmid (Fig 1). The recombinant plasmids were introduced into *Agrobacterium tumefaciens* strain EHA105. Expression of reporter β-glucuronidase (GUS) gene was measured in order to evaluate promoter activity.

### Generation and identification of transgenic plants

**Fig 2:**
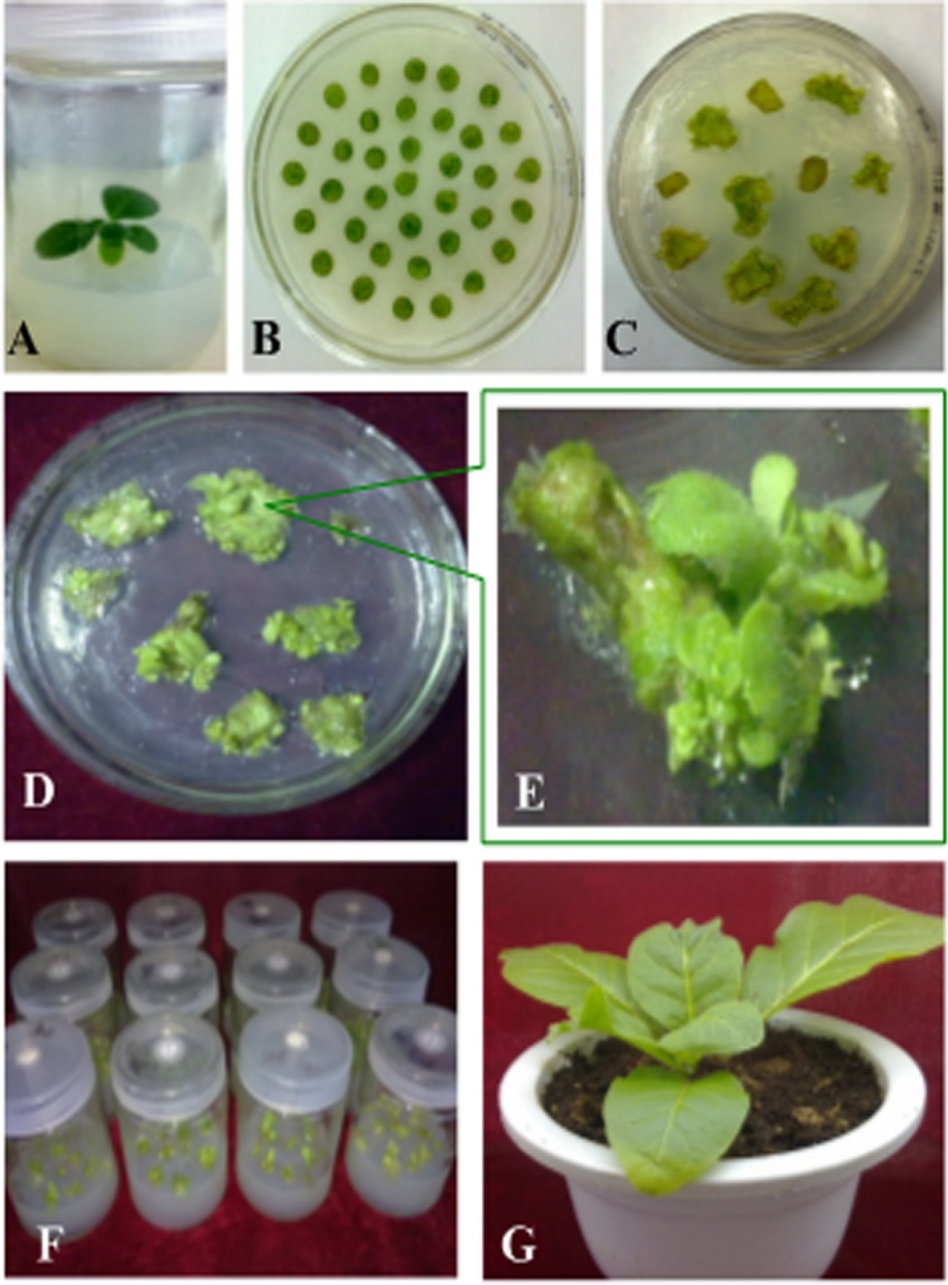
Generation of transgenic tobaccos. (A) Wild type tobacco; (B) Pre-culture tobaccos; (C) Selective culture transgenic tobaccos; (D) subculture transgenic tobaccos; (E) Screening of transgenic tobaccos resistant buds; (F) Rooting culture transgenic tobaccos; (G) transgenic tobacco seedlings.

Tobaccos were inflected using the Agrobacterium-mediated method. Briefly, the leaves were cut into small pieces and were cultured on MS premedium for 2 days followed by transgenic Agrobacterium tumefaciens strain EHA105 infection. The leaf pieces were cultured on selection medium and then were transferred onto rooting medium following growing sprouting, and finally potted in soil (Fig 2). The second generations of transgenic plants were used for the subsequent study.

**Fig 3:**
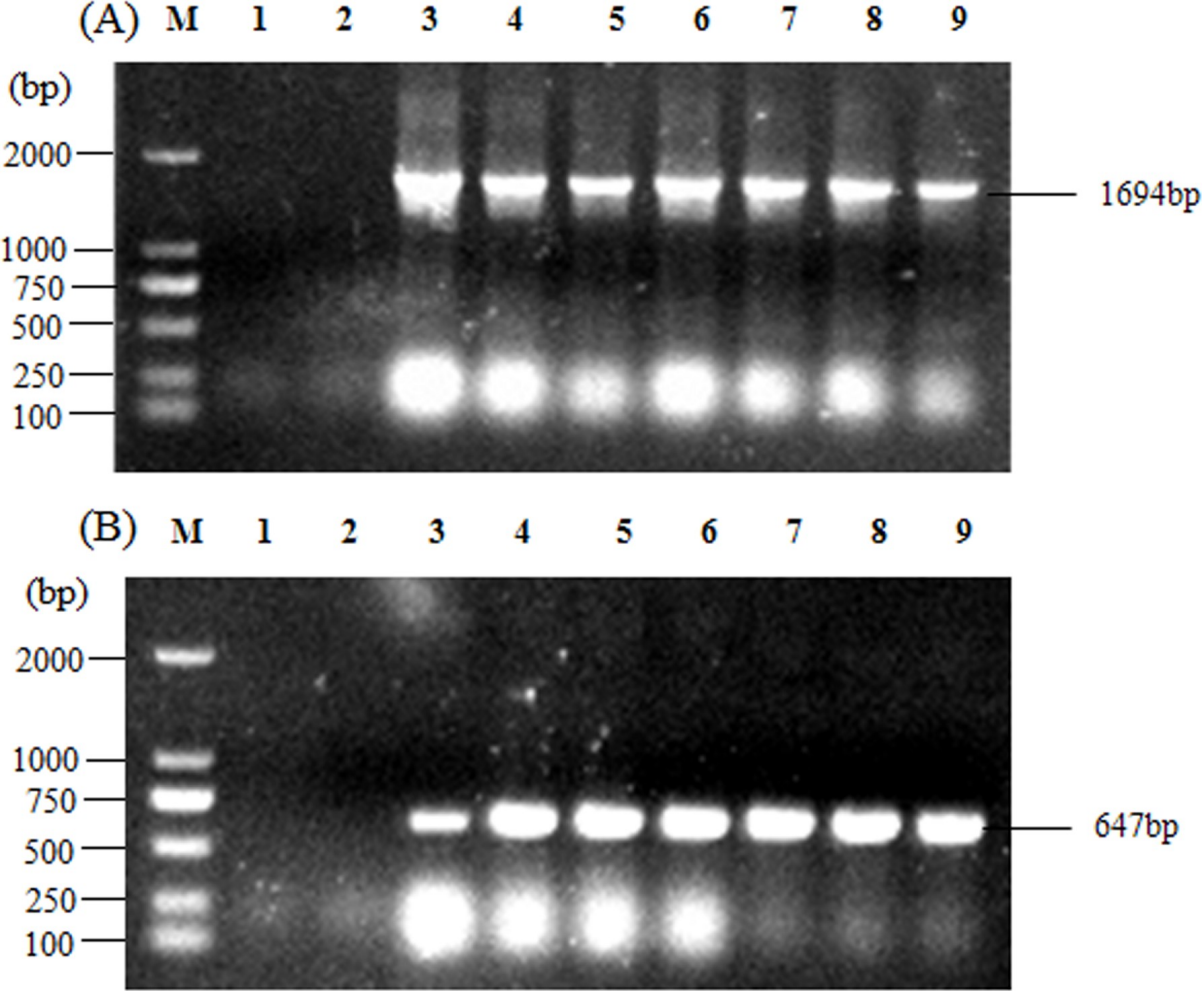
Identification of transgenic tobaccos. (A) *Zmap* promoter (1694 bp) in transgenic tobacco; (B) Hygromycin gene (647 bp) in transgenic tobacco; M: Marker, 1: water (blank control), 2: wild type tobacco (negative control), 3–9: transgenic tobaccos.

In total, 0.1g tobacco leaves were collected from each transgenic plant and the genome DNA were extracted using CTAB method. PCRs were carried out using the *Zmap* promoter and hygromycin gene contained in plasmid, with water as blank control and wild type tobacco as negative control (Fig 3).

As shown in Fig 3A, the *Zmap* promoter DNA fragment of 1694 bp can only be detected in seven transgenic tobaccos, while not in water or wild type tobacco controls. Similar results were observed in hygromycin gene plasmid PCR (Fig 3B). These results indicated that the target gene had been successfully transferred into transgenic tobaccos.

### Histochemical staining

Histochemical staining was performed as described previously [8]. Samples collected from transgenic tobacco after different stress-related stimuli were incubated in GUS reaction buffer (3 mg/ml X-gluc, 40 mM sodium phosphate pH7, 10mM EDTA, 0.1% Triton X-100, 0.5mM potassium ferricyanide, 0.5mM potassium ferrocyanide, and 20% methanol).Stained samples were bleached ethanol (70% (*v/v*)) to remove chlorophyll after overnight incubation (37°C) and observed under white light using a Nikon SMZ1000 microscope. GUS expression patterns in whole plants were visualized by histochemical assay.

### Plant treatment

The fourth and sixth leaves of the transgenic tobacco were used to investigate the effects of the different stress-related stimuli on GUS reporter gene expression. For drought stress treatment, tobacco plant roots were treated with 20% polyethylene glycol (PEG). Tobacco plants were put in growth chamber at 4°C for low temperature treatment. Tobacco plants were put in growth chamber at 4°C for low temperature treatment. Untransformed tobacco plants, plants transformed with CaMV35S (pCAMBIA1301 vector), and transgenic plants treated with water in the same areas were provided as controls. All tobacco samples were treated at 1, 3, 5, 10 and 24h. After each treatment, tobacco leaves were frozen in liquid nitrogen, and stored at a temperature of -80°C for total RNA isolation.

### Total RNA extraction and real-time quantitative RT-PCR analysis

Total RNA from tobacco leaves was extracted by the RNAiso Reagent (Takara, Changchun, China). Total RNA was reverse transcribed into single-stranded cDNA by using M-MLV Reverse Transcriptase and anoligo (T) 18 primer (Takara, Changchun, China). RT-PCR analysis was performed using SYBR Green I (TaKara) on an Applied Biosystems 7500real-time PCR machine (Applied Biosystems, Foster City, USA). The tobacco actingene (GenBank Accession No.U60491) was taken as endogenous control gene. RT-PCR primers are shown in Table 1. Real-time PCR cycling conditions were as follows: 95°C for 30sec; and 40 cycles of 95°C for 5s, 56°C for 40s. All reactions were performed three times. The data were analyzed via2^−ΔΔCT^ method [9].

### Fluorometric GUS assay

GUS activity was detected by fluorometric assay according to the method of Jefferson et al [8]. GUS extraction buffer (0.1% TritonX-100; 50 mM phosphate buffer, pH 7.0; 10 mM EDTA; 0.1% sodium lauryl sarcosine; 10 mM β-mercaptoethanol; 20% methanol) was used to extract various tissues of tobacco leaves. After centrifugation, the total protein content of extracted supernatants was measured by the Bradford method [10] by using a Bio Rad Protein Assay Kit with BSA as a standard. The assays were performed in triplicates for each sample.

## Results

### Structure analysis of *cis*-acting elements in *Zmap* promoter sequences

*Zmap* promoter was analyzed by bioinformatics using the PlantCARE database to identify the cis-acting regulatory elements. The 1694bp DNA sequence located upstream of the translation start site (indicated with “+1” at the ATG start codon of the *Zmap* gene) was considered the putative promoter in this study. Bioinformatic analysis of *Zmap* promoter allowed us to identify the existence of some putative regions that could modulate gene expression. These putative regions are also known as *cis*-acting regulatory elements (Table 2). *Zmap* promoter sequence contains several core fragments shown in Table 3. They consisted of one TGACG-motif (TGACG), one LTR(CCGAAA), one CGTCA-motif (CGTCA), one CAT-box (GCCACT), one box-II (TCCACGTGGC), one G-Box (GTGCAA), one GA-motif(TCATCTTT), three MYB binding sites (MBS, one TAACTG and two CAACTG), and many other cis-acting regulatory elements, such as TC-rich repeats, HD-Zip 2and AuxRR-core. Analysis of *Zmap* expression potentially indicates its regulation and expression by many different stress stimuli [11].

**Table 2:** Analysis of the *Zmap* promoter region.

Position	Sequence					
-1693	TGCCGTGATA	CCGACTTGAG	TCCGAAGGTA	CCTGCTCACA	CATTATACTT	CCAGAAATAC
					**TATA-box**	
-1633	TGTTAAATCC	TGTTTTTGAG	GACAGCAAAT	ATATTTAGAA	CCGACCCGTC	ACTATATTGT
-1573	AGTAGTGATG	TGGTCTGCAA	TTTTTTTTTA	TTTCTTCCAT	TTTTTGCATA	TAAACGTGCT
-1513	AGTGGTGTGG	ACGTGTGGTA	TGAATTTTTT	GGTTGTACTG	TGAATGAGAT	TGGACCTGTC
-1453	GCTCAGTGCA	ATGCGCTTAT	ATATCCACTA	AGATTGCTAT	TAACTGGTAA	TGCAGATCCA
					**MBS**	
-1393	ACTCACAGCA	CCTACGCACA	TCTACAATAG	AAAACGTCAT	CCGAAACACT	GTAGAGTCCA
				**TGACG-motif**	**LTR**	
-1333	GATCAATTTC	CCCACGGTGC	AAACATGGCA	CTATTGCTAG	CTGCATACTA	CAGAATTGAA
-1273	TAGTACAGCA	ACTATGATCC	CATCTAGGAA	TGACAGTGGT	AAGGTATATG	TAATTGGCGC
					**TATA-box**	
-1213	ACAATGTCAT	ACCCATACAT	ATTAGAGAAA	AATGTCTCAC	CCACTACATC	GTGGATACAA
-1153	AACTACGTCA	TACTCATACC	CACACCACCC	ACCACGGGTA	GTGGGTATCC	ATCGAATACC
	**CGTCA-motif**					
-1093	CATATATAAC	CACTAACATT	ACAATAGACA	CGATCAACAA	CATTCAACAT	TAAATAGCAA
-1033	CATCATACAA	GCCATGCATG	AGAGAGAACA	AGCCCCTTAA	TCTGGACTCA	TATGTTATAT
-973	GTTAACGGGT	CTCCCATCGA	GTAGCGGGTA	TTTGGCAAAA	GAGAACATGC	ACACATCCAT
-913	CATACCCAAT	GTATATAATA	AATGACCCAA	TAAAATACCT	ATAGGTATAA	AAAAACACCT
		**TATA-box**				
-853	TATATACATG	TGCACTAATA	GGTTTTTTTA	CCTATCAGAT	ATCGGGTTTC	AGGTATCCAC
	**TATA-box**		**TC-ricrepeats**			**box II**
-793	GTTGCCACCC	CTGATTCCCC	ATCTGTGTGG	CAGTTGTCTG	CAAAACCCAA	ATCCTGCACG
				**MBS**		
-733	AAACTGCATG	CATTTTAGGG	TAATATCACA	TGCATGCTTG	CATTTCATTG	GTTGGGTCTC
-673	TCCACTGCCA	CTCTCGACTC	GTCGAGACAG	AGAGCACTGG	GAAGCATGCA	CATGCTAAGT
	**CAT-box**					
-613	GCAGCACCAT	CAGTCCACAG	CCCCCGGCAT	CACATTAGTG	ACTCCACGGA	GCAAATAAAA
-553	GAGCCCTCGC	CACTCGCCAG	TGCTCCAATA	GCCCAGTTGC	CATCTCCCCC	GAGTGGTGCA
				**MBS**		
-493	GGCCAATCAT	TGTTTTTCAA	AAAAAAAAAC	TTTCTAACCG	CCGGAGATTA	GAGACCATTA
	**HD-Zip 2**					
-433	TTGCATGCTG	TGCAGGCCGC	AGCCGCCGGT	CACCCACTAG	CTATCGTCGC	ACCGAATTAG
-373	CCTAACCCGA	GGTAGTATTA	AGCTGTTTAG	TATGAGGAAT	GATCTAGTCC	ATCATCTTTT
						**GA-motif**
-313	CACTCCTCAC	TTTTTTTTGT	TTGGTTTGTG	GAATAAATTG	AGTTGATCAA	TCATCACCTC
-253	ATTCCTTATA	GTTATTTAGT	TAGTACTAAT	ATGAGAAATG	AGATCATCCC	ACCAAATTTG
-193	AGGAATGGAC	CTATGATGCA	CCACTATATT	TTGGATAAAG	TGATTCCTCA	AACCAAACAA
	**AuxRR-core**					
-133	CCCTATATTC		CGCGACGGAC	GATCGCTTTT	TACCGTCTAT	AAGAACAACG	ATGCAAGAAA
	**TATA-box**					
-73	CTGTGTGGAG	TGTGCAAGCT	CAATACAGGC	ACAGTGGAGA	TCGAGACAGC	TAGCAAGCAA
		**G-box**				
-13	CAAGCAAGCT	GAA***A***TG				
		+1				

Putative *cis*-acting regulatory elements, detected in the promoter fragment using the PlantCARE database, are indicated within grey shaded boxes. The translation start site is indicated with “+1”.

**Table 3:** Putative *cis*-acting elements and their positions in the *Zmap* promoter.

*Cis* element	Sequence	Position	Function
TC-rich repeats	GTTTTCTTAC	-832	defense and stress responsiveness element
TGACG-motif	TGACG	-1359	MeJA-responsiveness element
box II	TCCACGTGGC	-798	part of a light responsive element
LTR	CCGAAA	-1353	low temperature responsiveness element
HD-Zip 2	CAATCATTGTTTT	-490	leaf morphology development control element
AuxRR-core	GGTCCAT	-189	auxin responsiveness regulatory element
CAT-box	GCCACT	-667	meristem expression regulatory element
CGTCA-motif	CGTCA	-1148	MeJA-responsiveness regulatory element
G-Box	GTGCAA	-57	light responsiveness regulatory element
GA-motif	TCATCTTT	-315	part of a light responsive element
MBS	TAACTG	-1407	MYB binding site involved in drought-mediated induction
	CAACTG	-514; -757	

### GUS reporter gene expression from *Zmap* promoter in response to different stimuli

**Fig 4:**
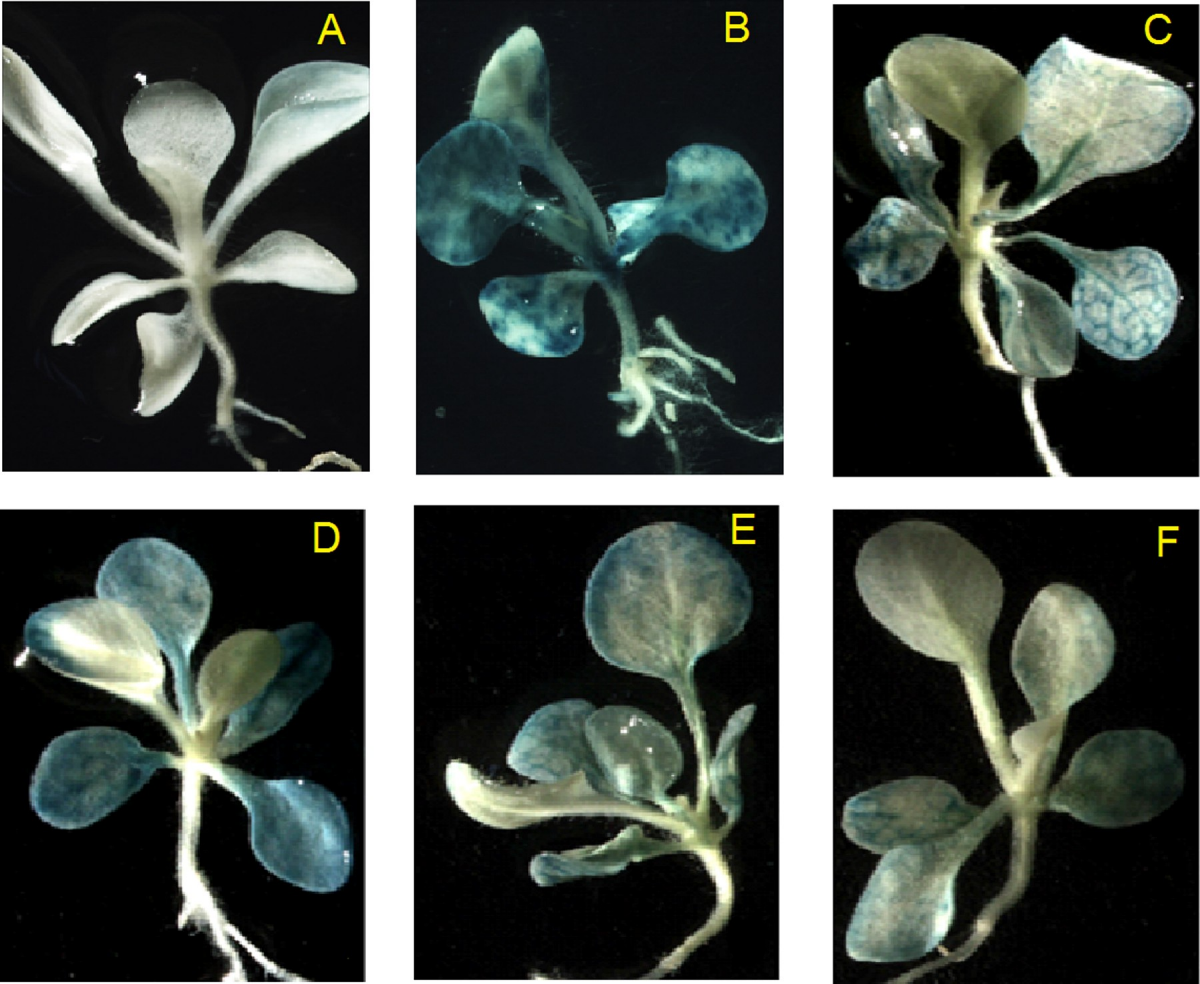
Histochemical staining of GUS activity in six-week-old transgenic tobacco plants. β-glucuronidase (GUS) expression in (**A**) wild-type; (**B**) CaMV35S-transformed tobacco plants; (**C**) untreated transgenic tobacco plants; and transformed tobacco plants treated with 20% polyethylene glycol (**D**), 100 μM methyl jasmonate (**E**) and low temperature (4°C) (**F**).

The 1694bp full-length *Zmap* promoter was transferred into tobacco plants following the fusion of the *GUS* reporter gene in a plant expression vector in order to determine the regulatory mechanisms of controlling the expression of *Zmap* gene. Histochemical GUS staining was used to measure the expression levels of GUS gene in transgenic tobacco which showed the inducible activity of the *Zmap* promoter. The data revealed that a decrease in *GUS* gene expression after low temperature treatment (4°C) (Fig 4A, 4C and 4F), but an increase after the other treatments (Fig 4A, 4C, 4D and 4E). Slight GUS staining was observed in untransformed plants, though these background levels were far below those observed in CaMV35S-transformed tobacco plants (Fig 4A, 4B and 4C). *GUS* gene expression occurred mainly in the aerial parts of the plants rather than the roots.

**Fig 5:**
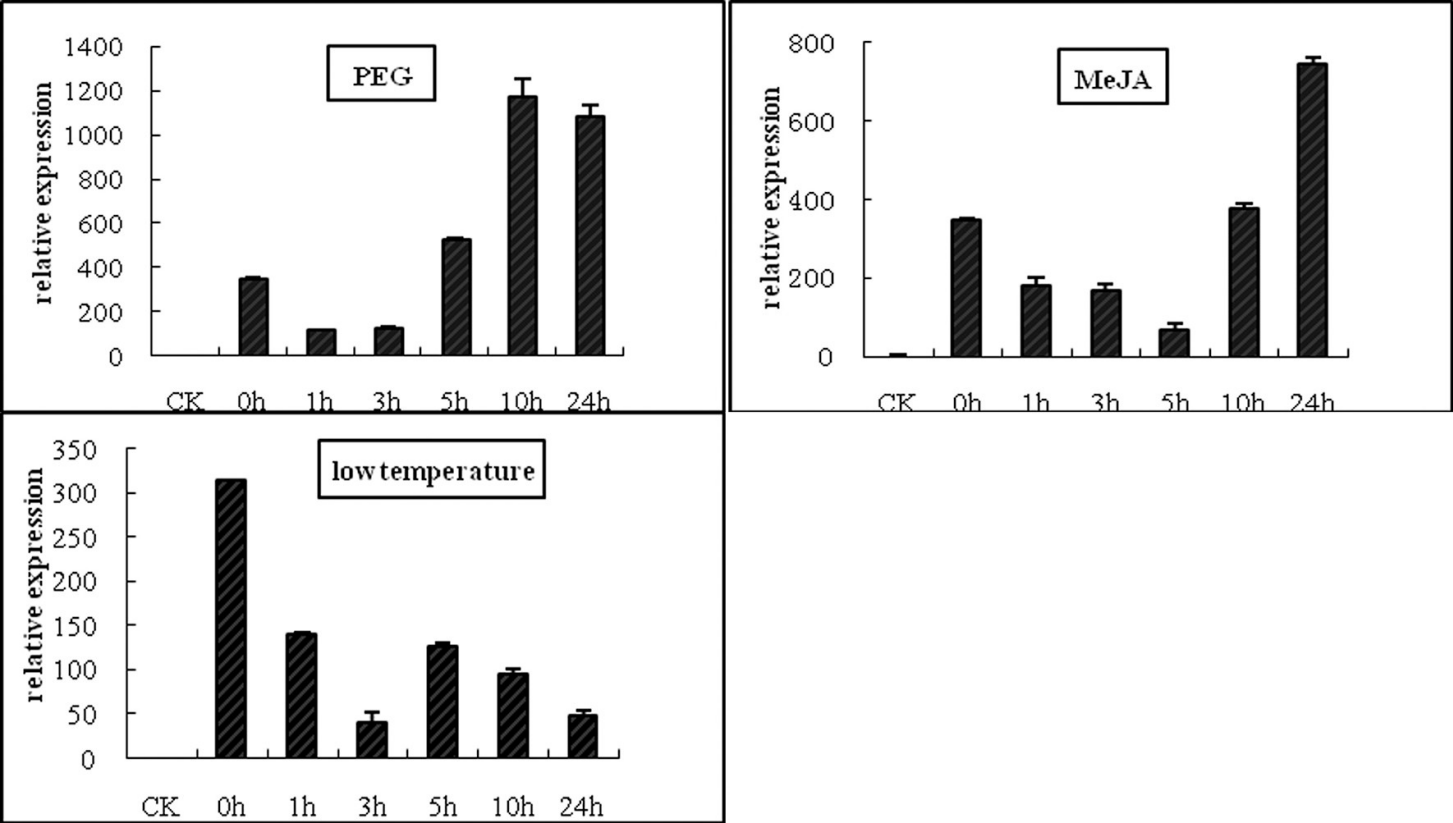
Time course of *GUS* transcript levels in the leaves of transgenic tobacco after treatment with polyethylene glycol (PEG), methyl jasmonate (MeJA) and low temperature (4°C). CK (wild-type) and 0 h-treated tobacco plants were left untreated as controls. At least three independent experiments were performed for each sample.

*GUS* reporter gene expression was examined quantitatively by real-time RT-PCR using total RNA extracted from the aerial parts of transgenic tobacco between the fourth and sixth leaves at chosen time points after treatment with MeJA, PEG, or low temperature (Fig 5). *GUS* transcript levels were induced by PEG, with a maximal level at 10 h. MeJA treatment also significantly increased *GUS* gene transcription at 24 h. In contrast, low temperature treatment decreased *GUS* transcript levels compared to the untreated control (0 h).

**Fig 6:**
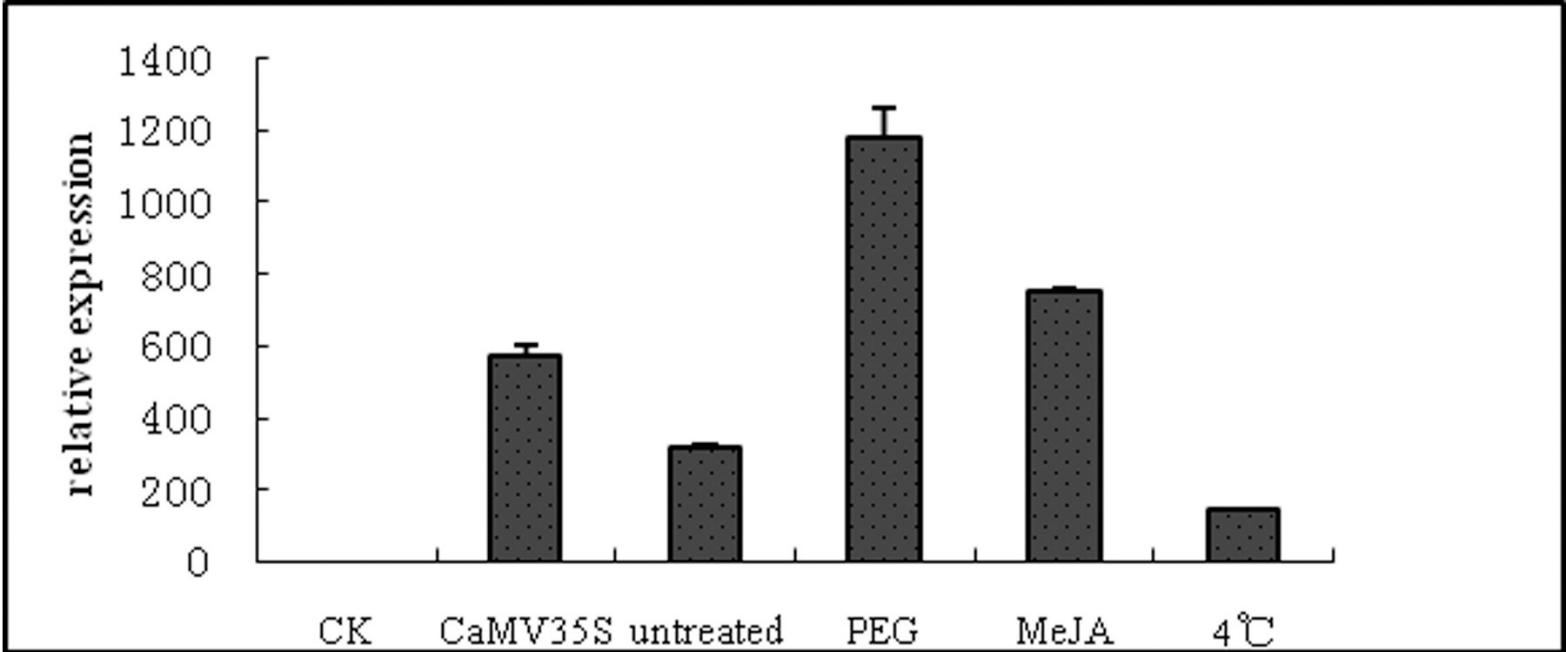
*Zmap* promoter-driven GUS activity after treatment with polyethylene glycol (PEG), methyl jasmonate (MeJA), and low temperature (4°C). GUS activity from CaMV35S (pCAMBIA1301 vector) transformants, wildtype, and untreated transformants served as controls.

Collectively, *GUS* expression levels were examined by histochemical GUS staining (Fig 6). The results confirmed our observations above, with GUS expression about 4-fold higher after PEG treatment. Expression levels were lowest in plants treated with low temperature, corroborating results showing inhibition of *GUS* gene expression in cold-treated transformed tobacco plants. MeJA-treated plants had 2-fold higher GUS expression levels compared to untreated plants. Differences in overall expression levels between the different treatments are probably due to differential rates of *gus* mRNA or protein turnover.

### Analysis of *Zmap* promoter deletion mutants

**Fig 7:**
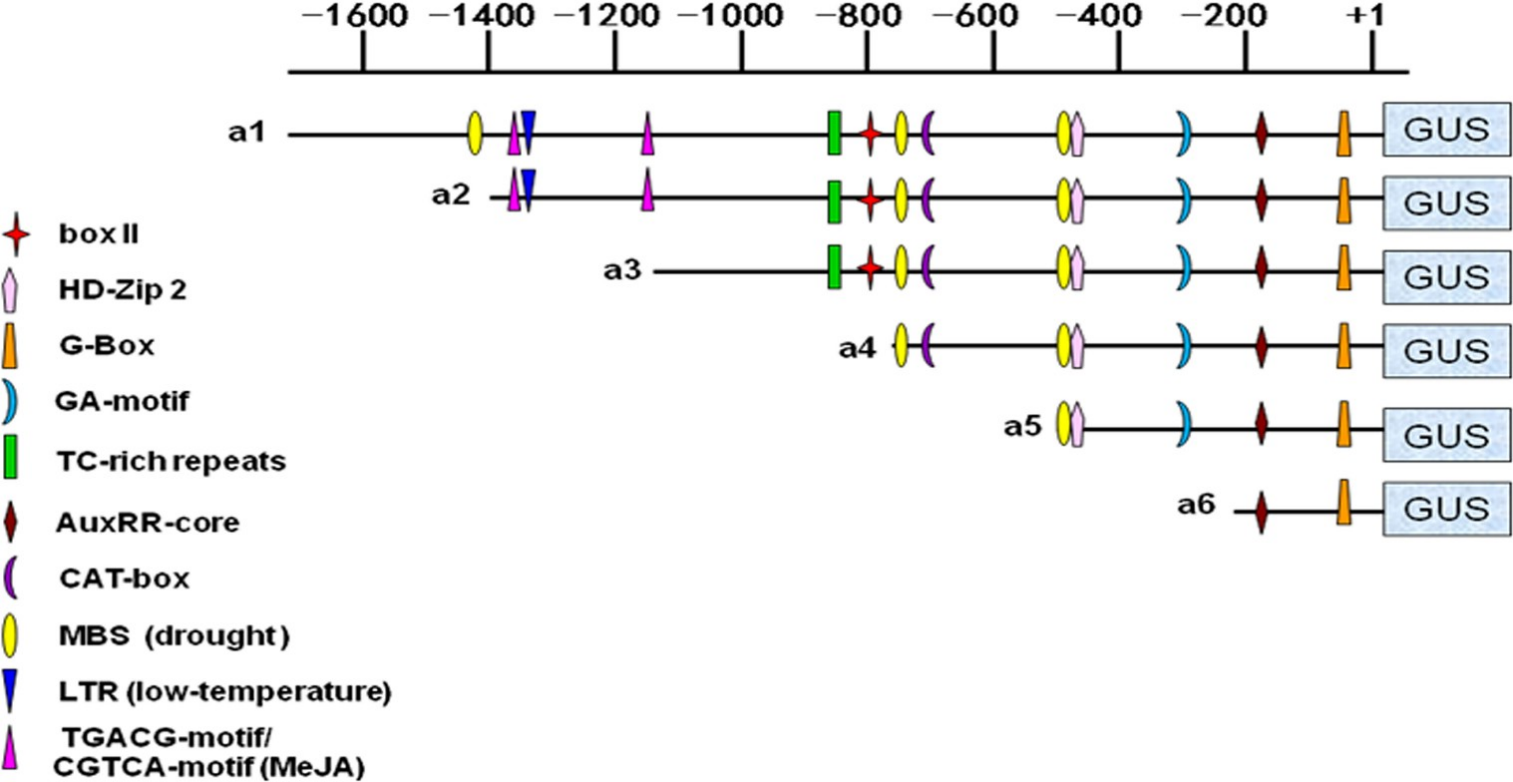
Bioinformatic analysis of *cis*-regulatory elements in the *Zmap* promoter and its six deletion fragments: a1 (1694 bp), a2 (1394 bp), a3 (1138 bp), a4 (784 bp), a5 (527 bp) and a6 (221 bp).

**Fig 8:**
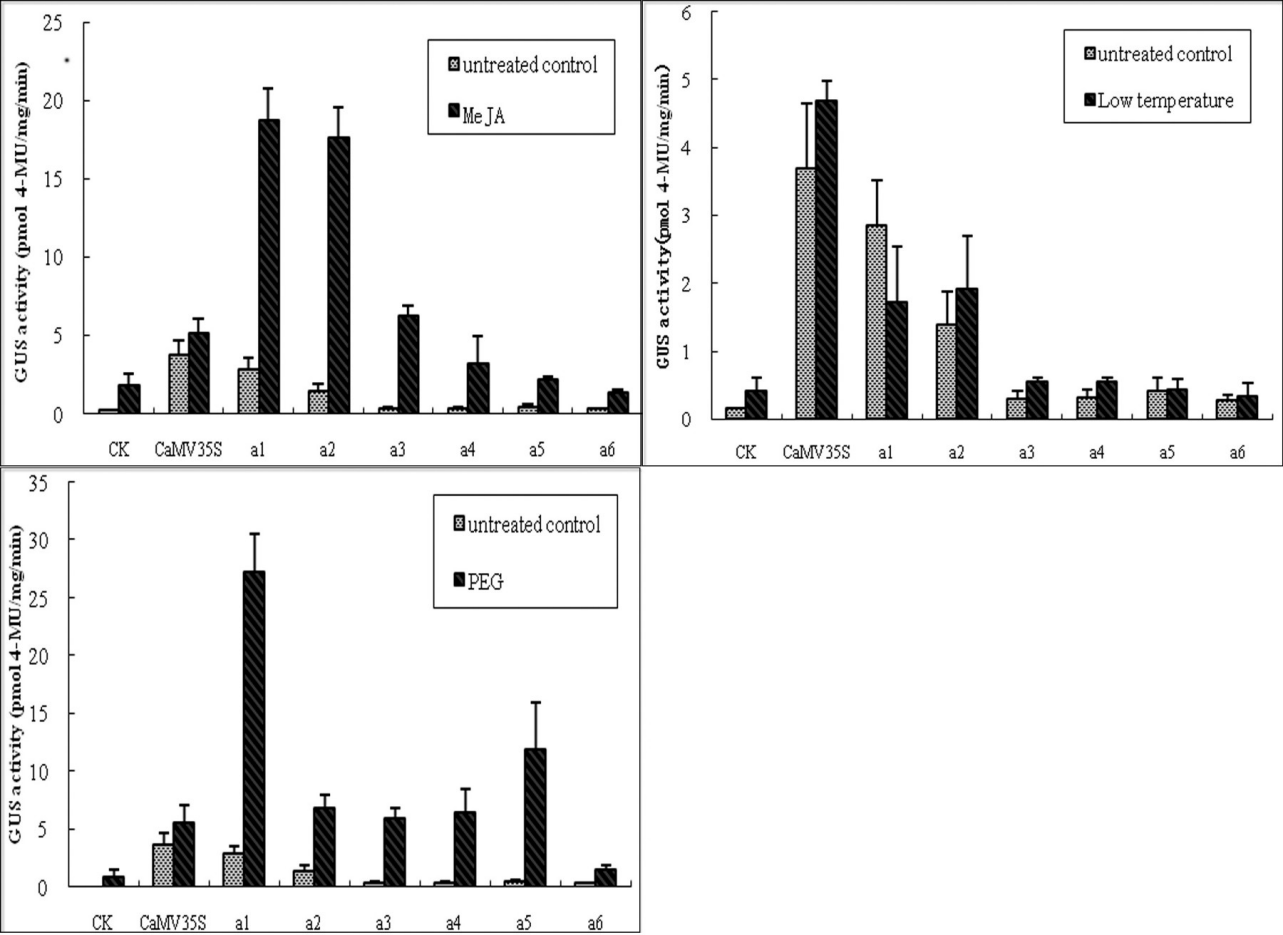
Analysis of *Zmap* promoter deletion mutants. GUS activities in plants carrying the *Zmap* promoter deletion series treated with low temperature (4°C), methyl jasmonate (MeJA) and polyethylene glycol (PEG). GUS activity from CaMV35S (pCAMBIA1301 vector) transformants, wild-type and untreated transformants served as controls.

To further study stress-inducible expression from the *Zmap* promoter, a series of 5′ promoter deletion-GUS constructs were transferred into the tobacco plant by transient expression. GUS expression in transformed plants was then measured by a fluorometric GUS assay. Deletion promoters were named a1 (1694 bp), a2 (1394 bp), a3 (1138 bp), a4 (784 bp), a5 (527 bp) and a6 (221 bp), respectively (Fig 7). Fluorometric GUS activity assay was used on the leaves of stress-treated transgenic tobaccos. It has been noted that GUS activity of a1-promoter plants was higher than other deletion promoter plants treated with phytohormone (MeJA) (Fig 8). Comparing with untreated controls, GUS activity of a1 and a2 deletion promoter plants increased significantly, suggesting that MeJA-responsive elements (TGACG-motif and CGTCA-motif) might play important roles on driving GUS expression in a1 and a2 plants. However, a1-mediated GUS activity was reduced significantly after low temperature treatment compared with untreated plants, while GUS expression increased in a2 plants (Fig 8). There was no obvious difference in GUS activity mediated by other deletion promoters. GUS activity increased in all plants after PEG treatment (Fig 8), with a1 plants increasing the most and a5 plants also showing a large increase.

## Discussion

Studies demonstrated that the expression of many plant genes such as, metabolic, regulatory and structural genes [12–15] were induced by light. In this study, the activity of the *Zmap* promoter was investigated by a fusion reporter construct (P*Zmap*: GUS) after transformation into the tobacco genome. GUS activity from transgenic plants provided a detailed pattern of *Zmap* promoter function. Leaves and stems of transgenic tobacco plants exhibited blue staining, but roots not showed any signs of blue staining (Fig 4). It was found that light could interfere with *Zmap* promoter, and several light-responsive elements such as the GA-motif, box-II and the G-box [16–18] were also found in the promoter sequence (Table 3).

Bioinformatic analysis revealed adversity stress elements (one low temperature element, two MeJA-responsive elements, and three putative drought-responsive elements) in the *Zmap* promoter region. These elements were hypothesized to have a strong effect on gene expression. To further understand the expression level of stress-induced transcriptional activity of the *Zmap* promoter at the protein level, real-time RT-PCR analysis was performed (Fig 5). *GUS* transcript levels decreased in response to low temperature treatment. In contrast, transcript levels increased in response to MeJA and drought treatment. These results were corroborated by histochemical GUS staining analysis (Fig 6).

Deletion analysis showed that the *Zmap* promoter possess adversity stress *cis*-regulatory elements that could allow maize to respond to stress. *Agrobacterium*-mediated leaf-disc was used to transform deletion promoter constructions into tobacco plants. In this study, GUS activity decreased with decreasing *Zmap* promoter length (a1—a6) in untreated plants. Interestingly, highest activity was shown the full-length *Zmap* promoter (a1) among all deletion promoters. It is speculated that *cis*-elements were found in the sequence between −1,694 and −1394bp of the *Zmap* promoter involved in up regulation of GUS expression.

Compared with untreated controls, the GUS activity of the a1 and a2 deletion promoter plants increased more significantly compared to other deletion promoter plants treated with MeJA (Fig 8). This result showed that the MeJA-responsive elements (TGACG-motif and CGTCA-motif) played a crucial role in enhancing the GUS activities of a1 and a2 [19–22]. Otherwise, a1-mediated GUS activity declined significantly under low temperature treatment, while a2-mediated activity increased in response to the same stimulus. No significant differences in GUS activity were found in other groups. It can be concluded that the low temperature responsive element has a positive regulatory role under low-temperature stimuli [23–24] and that there may be other still unidentified negative elements in this 300bp fragment (−1,694 to −1394). Sequence analysis showed that there are three drought responsive elements (MBS) in the *Zmap* promoter. Transcription factor MYB could bind to MBS, which could act as a target for other regulators [25–28]. After PEG treatment, a1 plants had the highest activity, followed by a5 plants. Therefore it can be obtained that these elements (-1407 and -514) play important roles under drought stimulation, while the (-757) element might act as a negative drought-responsive element.

In conclusion, the results revealed the activity patterns of the *Zmap* promoter, and thus could provide better understanding of the complex regulatory mechanisms and functional regions of *Zmap* promoter. A fluorometric GUS assay and qRT-PCR results indicated that *Zmap* promoter-mediated activity increased after MeJA and drought treatment but decreased after low temperature treatment. These data will support further studies of the role of adversity-inducible promoters in maize defense response and offer a foundation for improving the resistance of maize to different stressors.
